# Simultaneous Detection of Glucose and Fructose in Synthetic Musts by Multivariate Analysis of Silica-Based Amperometric Sensor Signals

**DOI:** 10.3390/s21124190

**Published:** 2021-06-18

**Authors:** Joaquin Rafael Crespo-Rosa, Giorgia Foca, Alessandro Ulrici, Laura Pigani, Barbara Zanfrognini, Laura Cubillana-Aguilera, José María Palacios-Santander, Chiara Zanardi

**Affiliations:** 1 Department of Analytical Chemistry, Faculty of Sciences, Campus de Excelencia Internacional del Mar (CEIMAR), Institute of Research on Electron Microscopy and Materials (IMEYMAT), University of Cadiz, Polígono del Río San Pedro S/N, 11510 Puerto Real, Cadiz, Spain; joakincrespo@hotmail.com (J.R.C.-R.); laura.cubillana@uca.es (L.C.-A.); josem.palacios@uca.es (J.M.P.-S.); 2 Department of Life Sciences, University of Modena and Reggio Emilia, via Amendola 2, 42122 Reggio Emilia, Italy; giorgia.foca@unimore.it (G.F.); alessandro.ulrici@unimore.it (A.U.); 3 Interdepartmental Research Centre, University of Modena and Reggio Emilia, BIOGEST-SITEIA, 42122 Reggio Emilia, Italy; laura.pigani@unimore.it; 4 Department of Chemical and Geological Sciences, University of Modena and Reggio Emilia, via G. Campi 103, 41125 Modena, Italy; 5 Institute for the Organic Synthesis and Photoreactivity (ISOF), National Research Council of Italy (CNR), Via P. Gobetti 101, 40129 Bologna, Italy; barbara.zanfrognini@isof.cnr.it

**Keywords:** sonogel-carbon, Au nanoparticles, composite, silica-based electrodes, amperometric sensors, glucose, fructose, principal component analysis, blind analysis

## Abstract

Silica-based electrodes which permanently include a graphite/Au nanoparticles composite were tested for non-enzymatic detection of glucose and fructose. The composite material showed an effective electrocatalytic activity, to achieve the oxidation of the two analytes at quite low potential values and with good linearity. Reduced surface passivation was observed even in presence of organic species normally constituting real samples. Electrochemical responses were systematically recorded in cyclic voltammetry and differential pulse voltammetry by analysing 99 solutions containing glucose and fructose at different concentration values. The analysed samples consisted both in glucose and fructose aqueous solutions at pH 12 and in solutions of synthetic musts of red grapes, to test the feasibility of the approach in a real frame. Multivariate exploratory analyses of the electrochemical signals were performed using the Principal Component Analysis (PCA). This gave evidence of the effectiveness of the chemometric approach to study the electrochemical sensor responses. Thanks to PCA, it was possible to highlight the different contributions of glucose and fructose to the voltammetric signal, allowing their selective determination.

## 1. Introduction

Sugars are essential organic compounds for both plants and humans, as they play several fundamental roles in their life and development. In particular, glucose and fructose are of main interest because they are essential nutrients in the human diet. They are naturally present in a wide range of food, or they may be added as additives.

The quantification of glucose and fructose is very important to evaluate the quality of many foodstuffs including honey, fruit and fruit juices. As an example, the simultaneous quantification of these two sugars is essential to determine the degree of ripeness of grapes; in oenology, this is a relevant factor as the characteristics of grapes directly affect the quality of wine. Due to the strong similarity between the chemical structures of the two carbohydrates, their quantification is generally performed after separation by chromatographic [1,2,3,4,5] or electrophoretic methods [6,7,8]. Despite allowing accurate quantification of both glucose and fructose in complex matrices, these analyses can only be carried out in a chemical laboratory. However, there are several situations requiring fast and reliable detection of sugar directly in-situ, e.g., during food production and storage or fruit maturation in the field. Amperometric sensors can be better suited to this purpose.

Although enzymatic biosensors can be exploited for the selective determination of glucose and fructose even in complex matrices [9,10], the use of devices based on biological recognition elements is not always possible. For example, such equipment cannot be used in case of high temperatures or high ionic strength or when aggressive chemicals, heavy metals or other compounds acting as enzymatic inhibitors are present. In addition, enzymatic biosensors can only be used as disposable devices, due to enzyme degradation in subsequent measurements. For this reason, there is a growing interest in the development of non-enzymatic electrochemical sensors, which allow the detection of carbohydrates with high sensitivity and long-term stability [11,12,13].

These devices generally take advantage of the electrocatalytic properties of transition metals, allowing efficient oxidation of sugars [14,15]. Recent works mainly deal with the development of sensors based on Cu and Ni derivatives [16,17,18,19,20,21,22,23]. However, these devices feature relatively high oxidation potential values (typically >0.4 V vs. Ag/AgCl), which can affect their selectivity when working in complex matrices, due to the possible oxidation of many interfering redox-active species. For this reason, more expensive Au-based sensors are often preferred.

Electrocatalytic oxidation of glucose at Au surfaces is one of the most discussed electrochemical processes in the scientific literature. The actual mechanism is, in fact, still quite debated. According to the most accepted theories [24,25] the electrocatalysis is achieved by the adsorption of glucose on the surface of the electrode and mediated by a hydrated oxide layer formed on the Au surface during the electrochemical oxidation process of the electrode surface. Even in more recent years, studies trying to explain the complex voltammetric responses normally obtained at polycrystalline structures of Au surfaces are still attracting the efforts of the electrochemical community [26,27]. In any case, it has been observed that the process is favoured in alkaline solutions as these conditions favour the formation of reactive sites on the electrode surface and of more easily oxidized reaction intermediate, namely enediol; moreover, OH^−^ species are required to neutralize the protons generated during sugars dehydrogenation steps. A very interesting aspect is that electrocatalytic activity towards glucose oxidation is preserved at Au nanostructured surfaces, with the advantage that the surface passivation is, in this case, limited [28].

The close similarity with the glucose molecule implies that the oxidation mechanism of the fructose molecule on the AuNPs can be explained by the same theories and mechanisms proposed in the case of glucose [24,29].

In previous work, we reported the performance of silica-based electrodes including a graphite-Au nanoparticles composite (C-AuNPs) [30]; they were synthesized by means of an innovative sonogel process [31]. The resulting SNG/C-AuNPs devices showed peculiar electrocatalytic activity toward glucose oxidation, reduced surface passivation and simple and reproducible surface renewal. All these characteristics make them suitable to be applied as electrochemical sensors for glucose determination. Since in several foodstuffs glucose is present together with fructose, in this research we tested this device for the simultaneous detection of both these sugars.

Firstly, aqueous solutions were considered, both to test the signal response of glucose and fructose oxidation separately from each other, and to evaluate the ability of the SNG/C-AuNPs device to distinguish the contribution deriving from the two sugars when analysing their mixtures. To this aim, since the electrochemical signals of the two sugars largely overlap, a multivariate approach based on Principal Component Analysis (PCA) was applied.

Then, based on the positive results obtained in the first step, a synthetic solution mimicking the composition of musts of red grapes was used instead of water to prepare a new set of samples obtained by adding variable amounts of glucose and fructose defined using a nine-level factorial design. This second set of samples was analysed to test the effectiveness of the device in a complex matrix containing other electroactive species such as polyphenol derivatives, which are acknowledged to induce passivation of the electroactive surfaces [32,33]. The dataset of electrochemical signals was once more explored by PCA, which allowed us to confirm the ability of the SNG/C-AuNPs electrode to detect the separate contribution of glucose and fructose concentration values.

## 2. Materials and Methods

All reagents by Sigma–Aldrich were of analytical grade and used without further purification. All solutions were prepared in Ultrapure water, obtained through a Milli-Q system (18 MΩ × cm resistivity, Millipore, Burlington, MA, USA).

**Figure 1:**
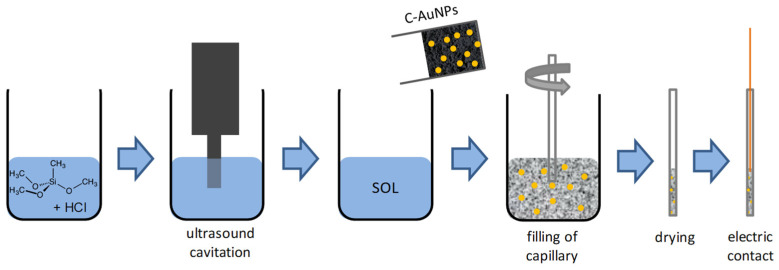
Scheme reporting the realization of SNG/C-AuNPs electrodes by following the approach described in [30,31].

SNG/C-AuNPs electrodes were obtained as reported in [30], starting from methyltrimethoxysilane, natural graphite powder (99.999 % pure, dimension < 74 μm) by Alfa-Aesar (Johnson Matthey GmbH, London, UK) and AuNPs encapsulated with citrate (16.3 nm mean diameter). They were made as schematized in Figure 1, by pouring 0.50 g of the C-AuNPs composite [30] in 0.6 mL of SNG solution; glass capillary tubes with 1.15 mm internal diameter were used as the body of the electrode.

Electrochemical measurements were carried out with an Autolab PGSTAT-30 (Eco Chemie, Utrecht, The Netherlands), under the control of GPES software. The electrochemical cell was completed by a Pt wire counter-electrode and an Ag/AgCl, 3 M KCl reference electrode.

All electrochemical tests were carried out in 17 mM KOH solutions at pH 12, also containing 0.1 M LiClO_4_ as the supporting electrolyte. Before each measurement, five consecutive voltammetric scans were performed in the pure electrolyte solution, to stabilise the background signal. No mechanical cleaning of the electrode surface was necessary between measurements in different solutions.

### 2.1. Electrochemical Tests in Aqueous Solutions of Glucose and Fructose

Each solution containing glucose and fructose was tested by recording both cyclic voltammetry (CV) and differential pulse voltammetry (DPV) in the potential interval −0.5–+0.6 V. As for CV tests, five consecutive scans were recorded at 0.05 Vs^−1^. In the end, the response of the last scan was taken into consideration since it represented the steady-state voltammogram. As for the DPV experiments, we adopted the following parameters: 50 mV modulation amplitude, 6 mV step potential, 0.1 s modulation time and 0.4 s interval time. As the DPV signals were collected just after having recorded the CV trace in the same solution, the first scan already consisted of the steady-state response.

Tests on a single analyte (either glucose or fructose) were carried out by consecutive addition of the analyte in the test solution and by recording both CV and DPV traces.

For preliminary tests in solutions containing a mixture of both the analytes, nine solutions were prepared considering a 3^2^ factorial design, i.e., testing all the possible combinations of the two analytes at the three concentration levels equal to 0.1, 1.0 or 1.9 mM, thus resulting in nine different solutions. In any case, the analysis in the presence of the analytes was performed after having recorded the signal in the blank electrolyte solution. Solutions with different composition were tested randomly to reveal the occurrence of possible memory effects.

A similar approach was also adopted to perform a further, more systematic set of measurements on mixtures of the two analytes, which were built based on a 9^2^ factorial design, i.e., considering all the possible combinations of nine equally spaced concentration levels of the two analytes, in the interval between 0 and 10 mM, which led to the 81 experimental conditions reported in Table 1. The repeatability of the sensor response was tested by including two further replicates of nine mixtures, highlighted in Table 1; in these cases, voltammetric responses almost overlapped. The resulting 99 mixtures (=81 experimental conditions + 18 replicates) were tested in random order.

### 2.2. Electrochemical Tests in Synthetic Must

Samples of synthetic red must were prepared to start from a solution simulating the composition of the real samples: 5 g/L tartaric acid, 1 g/L malic acid, 0.15 g/L citric acid, 500 mg/L tannins, 75 mg/L anthocyanin and adding different amounts of glucose and fructose. Based on the approach described previously for the systematic analysis of aqueous solutions, nine equally spaced concentration levels for each analyte, in the interval between 0 and 200 g/L were considered. They resulted in a set of mixtures characterized by the compositions reported in Table 2. This concentration range was defined based on the concentration range of sugars normally present in this matrix. In this case, the repeatability of the sensor response was tested by including two further replicates of nine mixtures, highlighted in Table 2.

The resulting 99 mixtures were diluted 100 times with 17 mM KOH (pH 12) solution, also containing 0.1 M LiClO_4_ as the supporting electrolyte. The concentrations of sugars of the final solutions were similar to those considered in the previous experimental design. The diluted samples were then tested by DPV and CV experiments, according to an order defined by a random number generator. In both cases, measurements in solutions in the presence of the analytes were alternated with measurements in the blank electrolyte solution. For this set of measurements, no mechanical cleaning step of the electrode surface was necessary.

### 2.3. Multivariate Analysis of CV and DPV Signals

The four datasets of signals considered for the chemometric treatment consisted of the CV and DPV responses recorded in the glucose and fructose mixtures (either aqueous solutions or synthetic musts) after subtraction of the relevant background signal, i.e., the signal collected in the electrolyte solution used for the preparation or dilution of the samples (17 mM KOH and 0.1 M LiClO_4_). The final responses were subjected to exploratory data analysis by means of Principal Component Analysis (PCA) (PLS-Toolbox, ver. 8.5, Eigenvector Research Inc., Wenatchee, WA, USA) running in MATLAB environment (ver. 9.3, The Mathworks Inc., Natick, MA, USA).

## 3. Results and Discussion

### 3.1. Electrochemical Responses of Fructose Oxidation

As discussed in [30], AuNPs in SNG/C-AuNPs are effective in activating electrocatalytic processes that allow the oxidation, thus the detection, of glucose. Although the oxidation of this analyte is also possible at neutral pH values with this electrode system, the sensitivity is higher in alkaline media. For this reason, the following tests were performed in solutions at pH 12, which represents the highest value for the electrode material to be stable. On the other hand, the concentration of sugars in real musts is so high that a dilution step before the analytical measurement is mandatory; this allows us to decide the most proper conditions at which performing the analytical measurements, consisting in a solution at pH 12 in our specific case.

**Figure 2:**
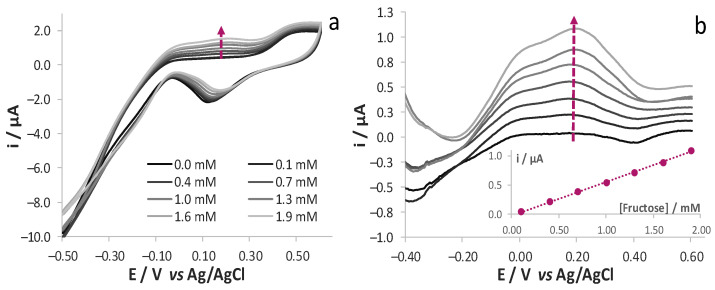
(**a**) CV responses registered at SNG/C-AuNPs electrode in solutions of 17 mM KOH and 0.1 M LiClO_4_ (pH 12) at different fructose concentrations; 0.05 Vs^−1^ potential scan rate and (**b**) the relevant forward traces subtracted of the background. Calibration plot obtained at +0.20 V are reported in the inset of (**b**).

SNG/C-AuNPs were tested in the same solvent media for the detection of fructose, which is the second main carbohydrate in must. AuNPs present in bulk electrode induced oxidation of fructose: CV responses showed (Figure 2a) the presence of an oxidation response both in the measurements from −0.5 to +0.6 V and vice versa, as expected for the oxidation of carbohydrates at gold surfaces [23,24,25,26,27]. Since the voltammetric responses also contain the contribution given by the oxidation of Au surface, they were subtracted from the response recorded in the absence of analyte. As better highlighted from the resulting forward scan (Figure 2b), the intensity of the oxidation peak at ca. +0.20 V linearly increases as the concentration of fructose within the explored range increases, i.e., between 0.1 and 1.9 mM.

**Figure 3:**
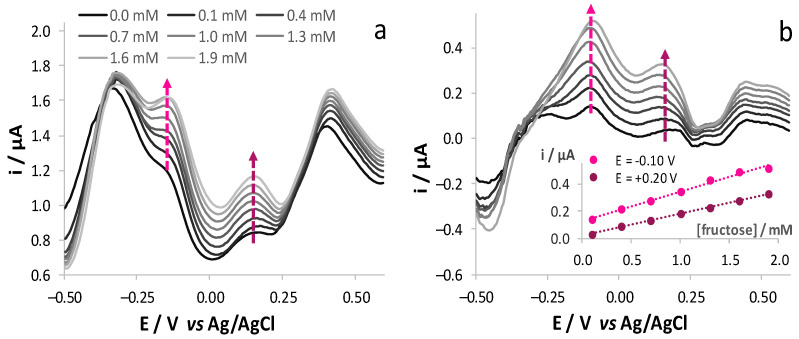
(**a**) DPV responses registered at SNG/C-AuNPs electrode in solutions of 17 mM KOH and 0.1 M LiClO_4_ (pH 12) at different fructose concentrations and (**b**) the relevant traces subtracted of the background. Calibration plot obtained at −0.10 V and +0.20 V is reported in the inset of *(***b**).

It is well known that CV is not the most proper electrochemical technique for analytical purposes, since it is affected by fairly high background and capacitive current values. A higher signal to noise ratio is generally achieved by recording DPV responses. This is the preferred voltammetric technique in electrochemical sensing. On the other hand, due to the quite peculiar electrochemical mechanism at the basis of glucose and fructose oxidation, which is quite far from being considered a pure diffusive one, the analysis is generally performed by the CV technique. To test the possibility of using this voltammetric technique for such an application, we recorded DPV responses in solutions at different concentration of fructose, once more evidencing a trend of the voltammetric signals with subsequent additions of fructose (Figure 3a). Better evidence of the contribution provided by fructose oxidation was once more obtained after background subtraction (Figure 3b): the intensity of a couple of peaks centred at ca. −0.10 and +0.20 V increases as the concentration of fructose in solution increases.

A similar trend was also observed for glucose oxidation, in this case resulting in a broad oxidation peak increasing from 0.0 to +0.2 V, similarly to what was observed in the CV responses reported previously [30].

### 3.2. Electrochemical Responses of Glucose and Fructose Mixtures

**Figure 4:**
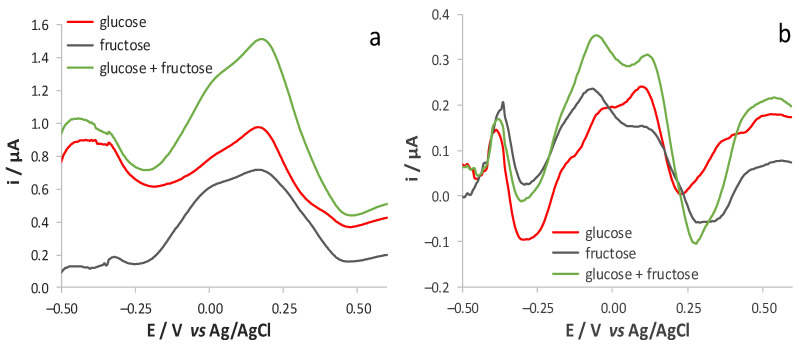
(**a**) Forward CV and (**b**) DPV responses obtained, after blank subtraction, at SNG/C-AuNPs electrode in 17 mM KOH and 0.1 M LiClO_4_ (pH 12) solutions containing either glucose or fructose (1.9 mM) or their mixture (1.9 mM glucose and 1.9 mM fructose).

Since complex oenological matrices contain a mixture of glucose and fructose, we tested the possibility to quantify the two analytes when they are present in the same matrix. Both CV (Figure 4a) and DPV (Figure 4b) traces obtained in a solution of either glucose or fructose showed characteristic peaks, whereas the responses recorded in a solution containing an equimolar ratio of the two analytes corresponds to the sum of the contributions of the two oxidation processes. As observed, the differences in the shape of the voltammetric responses of the two analytes are more evident when adopting DPV.

These results suggest the possibility to perform the selective determination of the two analytes by exploiting the different electrochemical behaviour of the two species. However, due to the strong overlap of the voltammetric responses ascribable to glucose and fructose oxidation, selectivity can be only reached by means of a chemometric treatment of the signals [34,35,36,37,38]. To test the effectiveness of this approach, we analysed the responses obtained from solutions containing both analytes in different molar ratios. As a first step, nine solutions containing different amounts of glucose and fructose (0.1, 1.0 and 1.9 mM) were prepared following a 3^2^ factorial design and they were analysed both by CV and by DPV. These solutions were tested in a randomized manner and the mechanical cleaning of the electrode surface was avoided to highlight the possible occurrence of memory effects. The nine responses were compared to investigate the effect of the increasing concentrations of either glucose or fructose in solutions containing a constant amount of the other analyte. The shape of the voltammograms was similar to the one of the pure analytes: as for the CV responses, the increase of fructose concentration with a constant amount of glucose induced the growth of the main peak at +0.20 V and a shoulder at ca. 0.0 V, whereas the increase of glucose concentration with a constant amount of fructose led to an increase of a main broad peak at +0.18 V. A similar behaviour was observed for DPV experiments, which led to responses qualitatively similar to those obtained from solutions of the single analytes, with the increase of the peak centred at ca. −0.15 and +0.15 V for fructose and glucose, respectively (Figure 4b).

These responses were also analysed from a quantitative point of view to evidence that the linearity of the sensor response for both glucose and fructose oxidation is maintained in solutions also containing either fructose or glucose, respectively. In addition, the value of the slope of the relevant calibration plots is not significantly different (α = 0.05) from each other, indicating that the presence of glucose does not affect the signal of fructose oxidation. An analogous evaluation was also carried out to prove the influence of the presence of fructose on the responses due to glucose oxidation. This led us to conclude that the simultaneous detection of the two analytes is feasible.

### 3.3. Multivariate Exploratory Data Analysis of Aqueous Solutions Containing Glucose and Fructose Mixtures

To perform a detailed characterization of the electrochemical signals measured on aqueous solutions of glucose and fructose mixtures, a more extensive set of measurements was performed on the mixtures created using the 9^2^ factorial design reported in Table 1. The reproducibility of the sensor response was tested by repeating the analysis three times in nine solutions at different composition (grey boxes in Table 1). This highlighted that almost overlapped voltammetric responses were achieved, especially when performing DPV analyses. In this case, the precision of the sensor was quantified by calculating the relative standard deviation (RSD) of the current values registered at the potential which had been previously identified as being typical of glucose and fructose oxidation. The average RSD values of the replicate measurements in these solutions resulted equal to 13.1% and 6.8% for the current values registered at +0.15 and −0.15 V, respectively.

A PCA model was then calculated for each one of the two datasets of CV and DPV signals to verify the ability of this multivariate approach to distinguish solutions containing different amounts of both sugars. The two PCA models were calculated using mean centring as preprocessing of the signals obtained after background subtraction that was performed to better identify the peaks of the two analytes. The results obtained by the two PCA models were very similar: in both cases, 3 principal components (PCs) were selected, whose explained variance was equal to 98.04% and to 94.81% for the models calculated on CV signals and DPV signals, respectively. Since the collection of DPV signals is a more rapid process, it led to more reproducible responses and highlighted more differences between the two analytes. For the sake of brevity, only the results of the PCA models calculated on the DPV dataset are reported and discussed.

**Figure 5:**
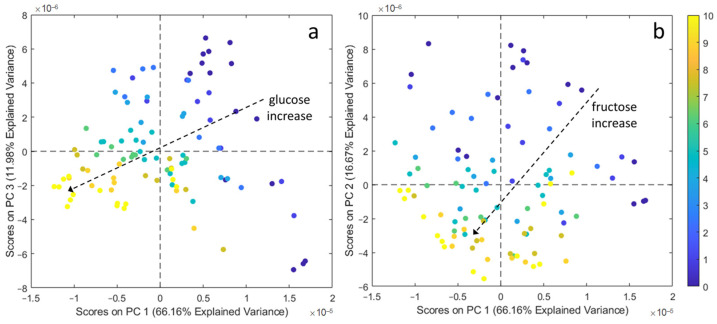
(**a**) PC1 vs. PC3 score plot with aqueous solution samples coloured according to the glucose content and (**b**) PC1 vs. PC2 score plot with aqueous solution samples coloured according to the fructose content. The colour bar refers to the sugar concentration which increases from 0 mM (blue) to 10 mM (yellow).

Figure 5a shows the PC1 versus PC3 score plot, where the aqueous solutions containing different amounts of glucose are represented with different shades of colour, ranging from blue to yellow with increasing sugar concentration, according to the values reported in the colour bar on the right part of the Figure. The increase of the glucose content resulted in the decreasing of both PC1 and PC3 values, following the direction highlighted by the dashed arrow. Considering the angles between the arrow and the two PCs, it is evident that the increase of glucose is mainly correlated with PC1: high glucose concentrations are found at negative PC1 values and vice versa. Figure 5b shows the score plot of PC1 versus PC2; in this case, the samples were coloured according to the fructose content. As indicated by the arrow, the fructose concentration increases as the values of both PCs decrease, with a major contribution by PC2. These results highlight that the DPV responses recorded in aqueous solutions reflect the concentration values of each individual analyte, regardless of the concentration of the other analyte, as it was previously hypothesised considering the preliminary analyses reported in Section 3.2 on a lower number of samples.

**Figure 6:**
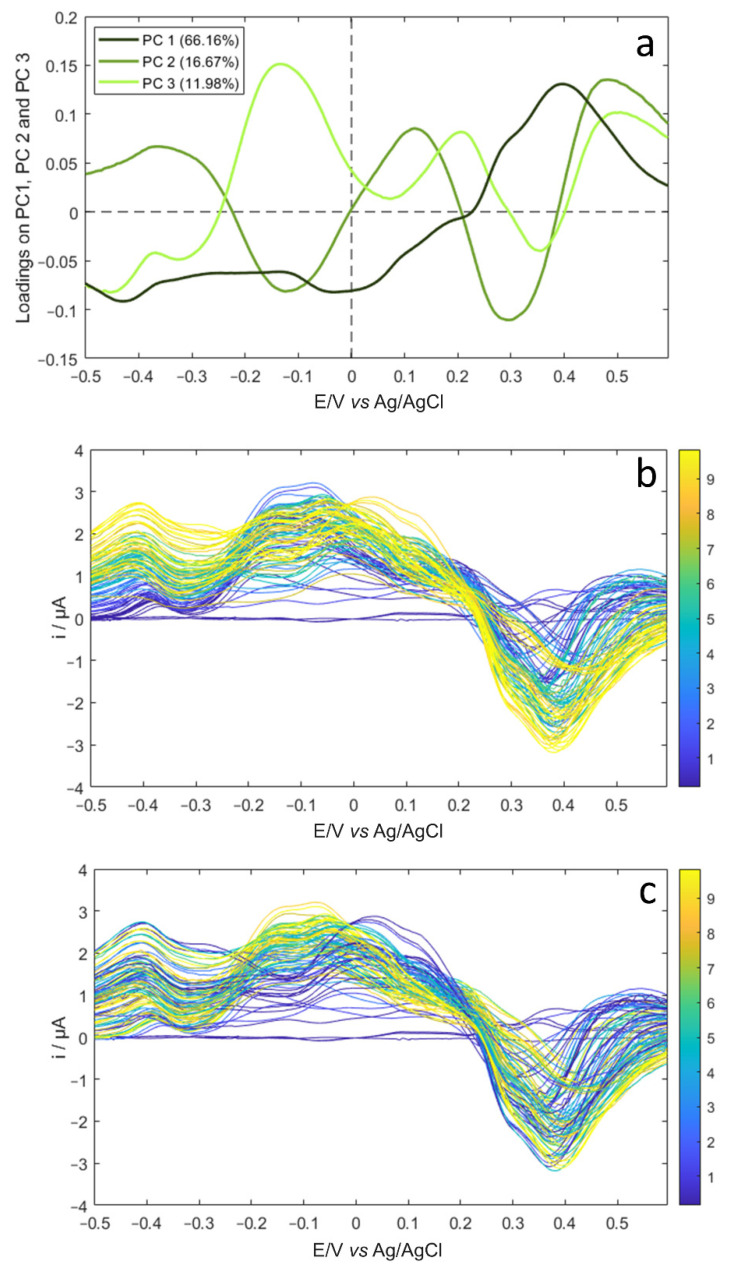
(**a**) Loading plots for PC1, PC2 and PC3 obtained by PCA on aqueous solution signals; (**b**) DPV signals coloured according to glucose content and (**c**) DPV signals coloured according to fructose content (the corresponding mM concentrations are reported in the colour bars).

To better describe the portions of the voltammetric signal that mostly contribute to the data structure observable in Figure 5, we reported the loading plots of the first three PCs (Figure 6a) compared to the voltammetric signals which have been coloured according to glucose (Figure 6b) and to fructose (Figure 6c) content.

Remarking that PC1 values decrease as the glucose concentration increases (Figure 5a), the comparison of the PC1 loading vector (dark green in Figure 6a) with the DPV signals, which have been coloured according to glucose concentration (from dark blue to yellow in Figure 6b), shows that the negative PC1 loading values in the range between −0.5 and +0.2 V correspond to the generally higher positive current values measured in this region for the mixtures with the higher concentration values. Similarly, the positive PC1 loading values in the signal region between +0.2 and +0.6 V correspond to the increase of the negative current values as the glucose concentrations increase. The origin of the positive contribution of glucose concentration to the current values in the potential range between −0.5 and +0.2 V is easily ascribable to the first step of glucose oxidation [39]. Conversely, it is difficult to give a ratio of the process occurring in the signal region between +0.2 and +0.6 V, where a current decrease is observed when passing from the blank electrolyte solution to solutions with increasing concentration values of glucose; this leads to solutions that contain a concentration of sugar higher than 2 mM, i.e., higher than those tested in Section 3.2, to the negative peaks centred at +0.4 V with a shoulder at about +0.3 V. However we need to remember that oxidation of glucose at Au surfaces occurs with a peculiar electrochemical process, where positive current values are normally recorded also in the scan toward negative potentials when reasonably high concentrations of sugars are analysed [25,30]. This can explain the negative values obtained in this potential region when adopting DPV as the investigation technique and after subtraction of the background signal from the response due to glucose/fructose oxidation.

Considering the PC2 and PC3 loading vectors (Figure 6a), the negative PC2 peak and the positive PC3 peak in the potential interval between −0.5 and +0.2 V correspond to the unique region where the contribution of fructose concentration is higher than the contribution of glucose concentration. In fact, the comparison of Figure 6b with Figure 6c in the potential region centred at about −0.15 V shows that the highest current values are obtained for the mixtures with a higher concentration of fructose. This result is consistent with the shape of the voltammetric responses reported in Figure 4b.

On the other hand, an opposite trend is observed in the potential region between 0 and +0.15 V, where the current values are higher for the solutions with a high glucose content and lower for the solutions with a high fructose content. This portion of the voltammetric signal contains the main oxidation peak ascribable to glucose oxidation, as was evident from Figure 4b.

### 3.4. Analysis of Glucose and Fructose Responses in Synthetic Musts

As a consequence of the promising results obtained with the PCA models calculated in aqueous solutions, the analysis was repeated in a more complex matrix mimicking the composition of real must samples, as described in Section 2.2.

**Figure 7:**
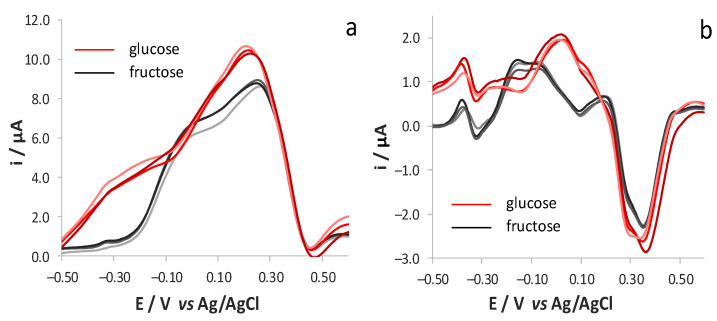
(**a**) Forward CV and (**b**) DPV responses registered, after blank subtraction, at SNG/C-AuNPs electrode in different solutions of synthetic red must contain either glucose or fructose (5 g/L) diluted 100 times with 17 mM KOH and 0.1 M LiClO_4_ (pH 12).

Basically, the shape of both CV and DPV responses obtained in the diluted must solutions (Figure 7) recalls those obtained in the pure electrolyte solutions of the sugars, evidencing that the interference due to other organic species present in the matrix are negligible when working at pH = 12. Figure 7 also shows that the responses recorded for the solutions possessing the same composition but which were analysed over different days have almost overlapped, even though no mechanical cleaning of the surface was performed. This demonstrates that fouling effects due to polyphenol oxidation are negligible, thanks to the electrochemical cleaning of the surface activated by the recording of the blank signal in a solution at pH 12, as reported in the Experimental section.

**Figure 8:**
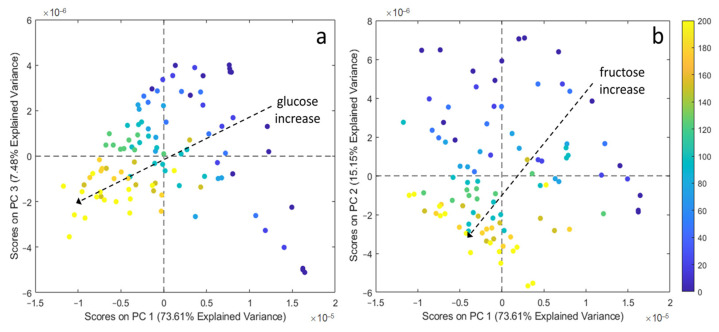
(**a**) PC1 vs. PC3 score plot of synthetic must samples coloured according to glucose concentration in the sample and (**b**) PC1 vs. PC2 score plot of synthetic must samples coloured according to fructose concentration in the sample. The colour bar refers to the sugar concentration which increases from 0 g/L (blue) to 200 g/L (yellow).

To demonstrate that glucose and fructose contributions to the electrochemical signals can be detected separately from each other also in samples possessing a more complex composition, the DPV and CV responses were evaluated using PCA in this case too. For reasons similar to those previously underlined, the discussion will only deal with the analysis of DPV responses. The PCA model calculated on the measured mean-centred DPV signals led to the selection of 3 PCs, accounting for 96.24% of total data variance. The score plots showing the data structure according to the concentration values of the two sugars are shown in Figure 8. It can be easily seen that Figure 5 and Figure 8 are very similar to each other, demonstrating that the electrode responses are minimally influenced by the interactions between the two sugars and the other chemical species that are present in the solutions used to mimic the synthetic must. The same behaviour described for the aqueous solutions is also observed in the loading plots of the first 3 PCs compared with the voltammetric signals of the synthetic musts. Once more this indicates that the contribution to the signal of the organic species that were added to mimic the composition of a must is almost negligible. Consequently, these results are encouraging given the application of the DPV technique to the quantification of glucose and fructose in food matrices in which they are simultaneously present.

## 4. Conclusions

The results here discussed demonstrated that the SNG/C-AuNPs electrode can be used as an effective sensor for the non-enzymatic detection of glucose and fructose in solutions at pH 12. The composite material containing AuNPs introduces effective electrocatalytic activities toward the oxidation of the two analytes; moreover the fouling effect, due to organic species normally present in real samples, does not affect the repeatability of the voltammetric responses. All these properties lead to a sensor showing oxidation responses for both glucose and fructose at quite low potential values and characterised by a repeatability which makes it suitable to be used in consecutive measurements of samples at different composition without a mechanical renewal of the surface.

Exploratory multivariate analysis of the signals, collected in samples containing both glucose and fructose in different molar ratios, demonstrated that the voltammetric response is the sum of the contributions of two analytes: no significant interference effects were observed in the response due to the oxidation of one of the two analytes when analysing solutions which contained the other analyte as well. The lack of electrochemical interaction between the two analytes, both in aqueous solution and in synthetic samples mimicking red musts, allows the correct identification of samples containing an increasing amount of either glucose or fructose, irrespective of the concentration of the second analyte. This fact proves the possible use of the same sensor for the simultaneous quantification of the two analytes in real samples. To this aim, tests on real musts samples are in progress in our laboratory; the very preliminary results confirm the effectiveness of the methodology that has been described hereinabove.

## Figures and Tables

**Table 1:** Composition of the 81 aqueous solutions (pH 12) of glucose and fructose, tested by CV and DPV; grey boxes indicate the samples prepared and tested three times, whereas white boxes lie for solutions tested only once.

	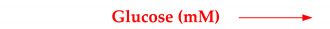								
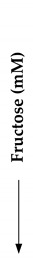	0.00–0.00	1.25–0.00	2.50–0.00	3.75–0.00	5.00–0.00	6.75–0.00	7.50–0.00	8.75–0.00	10.0–0.00
	0.00–1.25	1.25–1.25	2.50–1.25	3.75–1.25	5.00–1.25	6.75–1.25	7.50–1.25	8.75–1.25	10.0–1.25
	0.00–2.50	1.25–2.50	2.50–2.50	3.75–2.50	5.00–2.50	6.75–2.50	7.50–2.50	8.75–2.50	10.0–2.50
	0.00–3.75	1.25–3.75	2.50–3.75	3.75–3.75	5.00–3.75	6.75–3.75	7.50–3.75	8.75–3.75	10.0–3.75
	0.00–5.00	1.25–5.00	2.50–5.00	3.75–5.00	5.00–5.00	6.75–5.00	7.50–5.00	8.75–5.00	10.0–5.00
	0.00–6.75	1.25–6.75	2.50–6.75	3.75–6.75	5.00–6.75	6.75–6.75	7.50–6.75	8.75–6.75	10.0–6.75
	0.00–7.50	1.25–7.50	2.50–7.50	3.75–7.50	5.00–7.50	6.75–7.50	7.50–7.50	8.75–7.50	10.0–7.50
	0.00–8.75	1.25–8.75	2.50–8.75	3.75–8.75	5.00–8.75	6.75–8.75	7.50–8.75	8.75–8.75	10.0–8.75
	0.00–10.0	1.25–10.0	2.50–10.0	3.75–10.0	5.00–10.0	6.75–10.0	7.50–10.0	8.75–10.0	10.0–10.0

**Table 2:** Composition of the 81 solutions of synthetic red musts tested by CV ad DPV; grey boxes indicate the samples prepared and tested three times, whereas white boxes stand for solutions tested only once.

	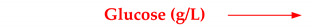								
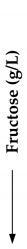	0–0	25–0	50–0	75–0	100–0	125–0	150–0	175–0	200–0
	0–25	25–25	50–25	75–25	100–25	125–25	150–25	175–25	200–25
	0–50	25–50	50–50	75–50	100–50	125–50	150–50	175–50	200–50
	0–75	25–75	50–75	75–75	100–75	125–75	150–75	175–75	200–75
	0–100	25–100	50–100	75–100	100–100	125–100	150–100	175–100	200–100
	0–125	25–125	50–125	75–125	100–125	125–125	150–125	175–125	200–125
	0–150	25–150	50–150	75–150	100–150	125–150	150–150	175–150	200–150
	0–175	25–175	50–175	75–175	100–175	125–175	150–175	175–175	200–175
	0–200	25–200	50–200	75–200	100–200	125–200	150–200	175–200	200–200

## Data Availability

Not applicable.

## References

[1] Paredes E., Maestre S.E., Prats S., Todolì J.L. (2006). Simultaneous Determination of Carbohydrates, Carboxylic Acids, Alcohols, and Metals in Foods by High-Performance Liquid Chromatography Inductively Coupled Plasma Atomic Emission Spectrometry. Anal. Chem..

[2] Ma C., Sun Z., Chen C., Zhang L., Zhu S. (2014). Simultaneous separation and determination of fructose, sorbitol, glucose and sucrose in fruits by HPLC-ELSD. Food Chem..

[3] Ghfar A.A., Wabaidur S.M., Ahmed A.Y.B.H., Alothman Z.A., Khan M.R., Al-Shaalan N.H. (2015). Simultaneous determination of monosaccharides and oligosaccharides in dates using liquid chromatography-electrospray ionization mass spectrometry. Food Chem..

[4] Filip M., Vlassa M., Coman V., Halmagyi A. (2016). Simultaneous determination of glucose, fructose, sucrose and sorbitol in the leaf and fruit peel of different apple cultivars by the HPLC–RI optimized method. Food Chem..

[5] Sun S., Wang H., Xie J., Su Y. (2016). Simultaneous determination of rhamnose, xylitol, arabitol, fructose, glucose, inositol, sucrose, maltose in jujube (*Zizyphus jujube* Mill.) extract: Comparison of HPLC–ELSD, LC–ESI–MS/MS and GC–MS. Chem. Centr. J..

[6] Cheng X., Zhang S., Zhang H., Wang Q., He P., Fang Y. (2018). Determination of carbohydrates by capillary zone electrophoresis with amperometric detection at a nano-nickel oxide modified carbon paste electrode. Food Chem..

[7] Tuma P., Málková K., Samcová E., Stulík K. (2011). Rapid monitoring of mono- and disaccharides in drinks, foodstuffs and foodstuff additives by capillary electrophoresis with contactless conductivity detection. Anal. Chim. Acta.

[8] Dominguez M.A., Jacksén J., Emmer Å., Centurión M.E. (2016). Capillary electrophoresis method for the simultaneous determination of carbohydrates and proline in honey samples. Microchem. J..

[9] Antiochia R., Palleschi G. (1997). A Tri-Enzyme Electrode Probe for the Sequential Determination of Fructose and Glucose in the Same Sample. Anal. Lett..

[10] Vargas E., Gamella M., Campuzano S., Guzmán-Vázquez de Prada A., Ruiz M.A., Reviejo A.J., Oingarron J.M. (2013). Development of an integrated electrochemical biosensor for sucrose and its implementation in a continuous flow system for the simultaneous monitoring of sucrose, fructose and glucose. Talanta.

[11] Toghill K.E., Compton R.G. (2010). Electrochemical non-enzymatic glucose sensors: A perspective and an evaluation. Int. J. Electrochem. Sci..

[12] Wang G., He X., Wang L., Gu A., Huang Y., Fang B., Geng B., Zhang X. (2013). Non-enzymatic electrochemical sensing of glucose. Microchim. Acta.

[13] Hwang D.W., Lee S., Seo M., Dong Chung T. (2018). Recent advances in electrochemical non-enzymatic glucose sensors. A review. Anal. Chim. Acta.

[14] Si P., Huang Y., Wang T., Ma J. (2013). Nanomaterials for electrochemical non-enzymatic glucose biosensors. RSC Adv..

[15] Terzi F., Pigani L., Zanardi C. (2019). Unusual metals as electrode materials for electrochemical sensors. Curr. Opin. Electrochem..

[16] Liu X.W., Pan P., Zhang Z.M., Guo F., Yang Z.C., Wei J., We Z. (2016). Ordered self-assembly of screen-printedflower-like CuO and CuO/MWCNTs modified graphite electrodes and applications innon-enzymatic glucose sensor. J. Electroanal. Chem..

[17] He J., Zhong Y., Xu Q., Sun H., Zhou W., Shao Z. (2018). Nitrogen-Doped Graphic Carbon Protected Cu/Co/CoO Nanoparticles for Ultrasensitive and Stable Non-Enzymatic Determination of Glucose and Fructose in Wine. J. Electrochem. Soc..

[18] Guellis C., Valério D.C., Bessegato G.G., Boroski M., Dragunski J.C., Lindino C.A. (2020). Non-targeted method to detect honey adulteration: Combination of electrochemical and spectrophotometric responses with principal component analysis. J. Food Comp. Anal..

[19] Wu H., Tian Q., Zheng W., Jiang Y., Xu J., Li X., Zhang W., Qiu F. (2019). Non-enzymatic glucose sensor based on molecularly imprinted polymer: A theoretical, strategy fabrication and application. J. Sol. St. Electrochem..

[20] Jeevanandham G., Jerome R., Murugan N., Preethika M., Vediappan M., Sundramoorthy A.K. (2020). Nickel oxide decorated MoS2 nanosheet-based non-enzymatic sensor for the selective detection of glucose. RSC Adv..

[21] López-Fernández E., Gil-Rostra J., Espinós J.P., Gonzalez R., Yubero F., de Lucas-Consuegra A., González-Elipe A.R. (2020). Robust label-free Cu_x_Co_y_O_z_ electrochemical sensors for hexose detection during fermentation process monitoring. Sens. Act. B Chem..

[22] Revenga-Parra M., Robledo S.N., Martínez-Periñán E., González-Quirós M.M., Colina A., Heras A., Pariente F., Lorenzo E. (2020). Direct determination of monosaccharides in honey by coupling a sensitive new Schiff base Ni complex electrochemical sensor and chemometric tools. Sens. Act. B Chem..

[23] Pérez-Fernández B., Martín-Yerga D., Costa-García A. (2017). Galvanostatic electrodeposition of copper nanoparticles on screen-printed carbon electrodes and their application for reducing sugars determination. Talanta.

[24] Pletcher D. (1984). Electrocatalysis: Present and future. J. Appl. Electrochem..

[25] Burke L.D., Ryan T.G. (1992). The role of incipient hydrous oxides in the oxidation of glucose and some of its derivatives in aqueous media. Electrochim. Acta.

[26] Pasta M., La Mantia F., Cui Y. (2010). Mechanism of glucose electrochemical oxidation on gold surface. Electrochim. Acta.

[27] Arjona N., Trejo G., Lodesma-García J., Arriaga L.G., Guerra-Balcázar M. (2016). An electrokinetic-combined electrochemical study of the glucose electro-oxidation reaction: Effect of gold surface energy. RSC Adv..

[28] Tominaga M., Shimazoe T., Nagashima M., Taniguchi I. (2005). Electrocatalytic oxidation of glucose at gold nanoparticle-modified carbon electrodes in alkaline and neutral solutions. Electrochem. Commun..

[29] Burke L.D. (1994). Premonolayer oxidation and its role in electrocatalysis. Electrochim. Acta.

[30] Crespo Rosa J.R., Zanardi C., Elkaoutit M., Terzi F., Seeber R., Naranjo Rodriguez I. (2014). Electroanalytical applications of a graphite-Au nanoparticles composite included in a sonogel matrix. Electrochim. Acta.

[31] Del Mar Cordero-Rando M., Hidalgo-Hidalgo de Cisneros J.L., Blanco E., Rodríguez I. (2002). The Sonogel-Carbon Electrode As a Sol−Gel Graphite-Based Electrode. Anal. Chem..

[32] Della Pelle F., Rojasa D., Scroccarello A., Del Carlo M., Ferraro G., Di Mattia C., Martuscelli M., Escarpa A., Compagnone D. (2019). High-performance carbon black/molybdenum disulfide nanohybrid sensor for cocoa catechins determination using an extraction-free approach. Sens. Act. B Chem..

[33] Pigani L., Seeber R., Bedini A., Dalcanale E., Suman E. (2014). Adsorptive-Stripping Voltammetry at PEDOT-Modified Electrodes. Determination of Epicatechin. Food Anal. Methods.

[34] Pigani L., Vasile Simone G., Foca G., Ulrici A., Masino F., Cubillana-Aguilera L., Calvini R., Seeber R. (2018). Prediction of parameters related to grape ripening by multivariate calibration of voltammetric signals acquired by an electronic tongue. Talanta.

[35] Pigani L., Rioli C., Foca G., Ulrici A., Seeber R., Terzi F., Zanardi C. (2016). Determination of polyphenol content and colour index in wines through PEDOT-modified electrodes. Anal. Bioanal. Chem..

[36] Pigani L., Culetu A., Ulrici A., Foca G., Vignali M., Seeber R. (2011). Pedot modified electrodes in amperometric sensing for analysis of red wine samples. Food Chem..

[37] Palacios-Santander J.M., Cubillana-Aguilera L.M., Cocchi M., Ulrici A., Naranjo-Rodríguez I., Seeber R., Hidalgo-Hidalgo de Cisneros J.L. (2008). Multicomponent analysis in the wavelet domain of highly overlapped electrochemical signals: Resolution of quaternary mixtures of chlorophenols using a peg-modified Sonogel-Carbon electrode. Chemom. Intell. Lab. Syst..

[38] Martina V., Ionescu K., Pigani L., Terzi F., Ulrici A., Zanardi C., Seeber R. (2007). Development of an electronic tongue based on a PEDOT-modified voltammetric sensor. Anal. Bioanal. Chem..

[39] Terzi F., Zanfrognini B., Zanardi C., Pigani L., Seeber R. (2011). Poly(3,4-ethylenedioxythiophene)/Au-nanoparticles composite as electrode coating suitable for electrocatalytic oxidation. Electrochim. Acta.

